# Lignan Glucosides from the Stem Barks of *Illicium difengpi*

**DOI:** 10.3390/molecules21050607

**Published:** 2016-05-10

**Authors:** Zheng-Hong Pan, De-Sheng Ning, Si-Si Huang, Ling Cheng, Meng-Wen Xia, Li-Yan Peng, Dian-Peng Li

**Affiliations:** 1 Guangxi Key Laboratory of Functional Phytochemicals Research and Utilization, Guangxi Institute of Botany, Chinese Academy of Sciences, Guilin 541006, China; ndshgxib@sina.com (D.-S.N.); ssh520123@163.com (S.-S.H.); 15507837860@163.com (L.C.); xmw1036978486@126.com (M.-W.X.); 2 Faculty of Chemistry and Chemical Engineering, Yunnan Normal University, Kunming 650500, China; 3 State Key Laboratory of Phytochemistry and Plant Resources in West China, Kunming Institute of Botany, Chinese Academy of Sciences, Kunming 650204, China; pengliyan@mail.kib.ac.cn

**Keywords:** *Illicium difengpi*, stem barks, lignan glucosides, difengpiosides A–D, NO inhibition

## Abstract

In this study, four new lignan glucosides, named difengpiosides A–D (**1**–**4**), were isolated from the stem barks of *Illicium difengpi*, together with seven known compounds **5**–**11**. Their structures were identified on the basis of spectroscopic analyses (1D and 2D NMR, HRESIMS, CD) and a comparison with literature data. All the compounds were evaluated for their inhibitory effects on lipopolysaccharide (LPS)-induced nitric oxide (NO) production in RAW264.7 cells.

## 1. Introduction

*Illicium difengpi* K. I. B et K. I. M. (Illiciaceae), native to China, is a toxic shrub that grows in the mountainous areas of Guangxi Province. Its stem bark is listed in the Chinese Pharmacopeia as a traditional Chinese medicine to treat rheumatic arthritis [1]. Previous phytochemical studies on this plant mainly focused on the low and middle polarity components and reported the isolation of over 40 compounds, including phenylpropanoids, lignans, triterpene acids, sesquiterpenes and others [2,3,4,5,6,7]. However, there are few investigations on the polar substances of *I.* *difengpi*. In order to provide comprehensive chemistry support for pharmacological studies and quality control of *I. difengpi*, the present study describes the isolation and structure elucidation of four new lignan glycosides together with seven known compounds from the *n*-BuOH-soluble fraction of an EtOH extract of the stem barks of this plant, as well as their inhibitory activities against nitric oxide production in lipopolysaccharide-induced RAW264.7 cells.

## 2. Results and Discussion

**Figure 1:**
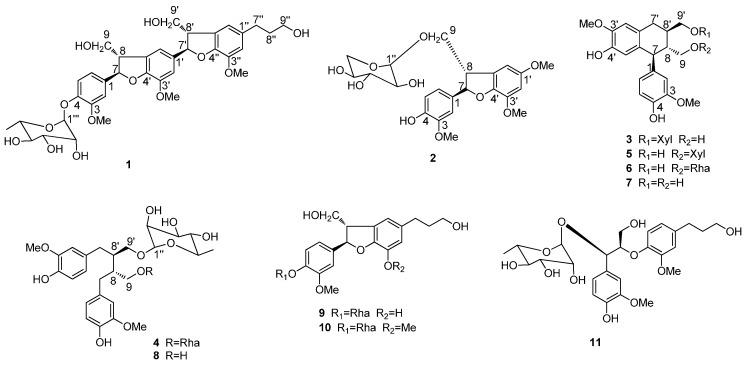
Chemical structures of 1–11.

The EtOH extract of the stem barks of *I. difengpi* were suspended in water and successively partitioned with petroleum ether, EtOAc, and *n*-BuOH. The *n*-BuOH-soluble extract was subjected to multiple chromatographic fractionations using silica gel, Sephadex LH-20, and ODS, yielding compounds **1**−**11** (Figure 1).

**Figure 2:**
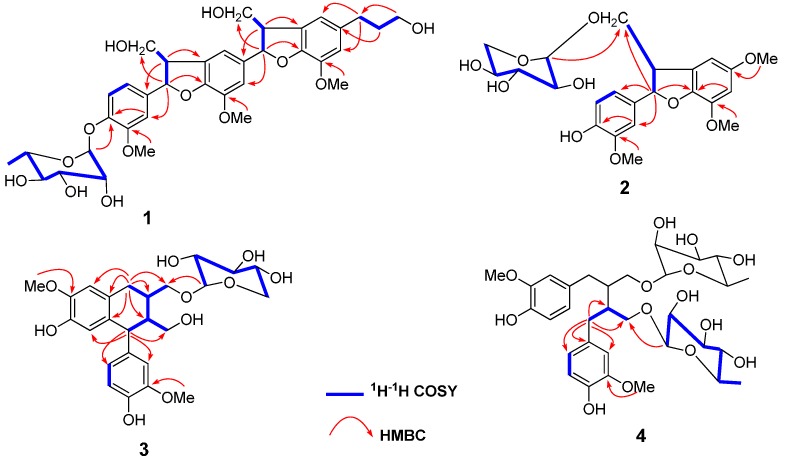
Key ^1^H-^1^H COSY and HMBC correlations of compounds **1**−**4**.

Compound **1** was obtained as an amorphous powder. The molecular formula was determined as C_36_H_44_O_13_ on the basis of a HRESIMS peak at *m*/*z* 707.2623 [M + Na]^+^ (calcd. for 707.2674), indicating 15 degrees of unsaturation. The IR spectrum showed the presence of hydroxyl (3406 cm^−1^) and aromatic ring (1595 and 1501 cm^−1^) groups. The ^1^H-NMR spectrum (Table 1) displayed signals at *δ*_H_ 7.07 (1H, d, *J* = 8.5 Hz), 7.01 (1H, d, *J* = 2.0 Hz) and 6.89 (1H, dd, *J* = 8.5, 2.0 Hz) for an AMX aromatic spin system, and four broad singlets at *δ*_H_ 6.92 (2H) and 6.72 (2H) for two tetrasubstituted aromatic rings, as well as three aromatic methoxy groups at *δ*_H_ 3.85 (6H, s) and 3.80 (3H, s). The remaining signals indicated the presence of a sugar moiety, an *n*-propanol and two −O−CH−CH−CH_2_O− spin systems, which were also confirmed by the ^1^H-^1^H COSY spectrum (Figure 2). The ^13^C-NMR spectrum (Table 1) showed the presence of six characteristic rhamnopyranoside signals (*δ*_C_ 101.5, 73.9, 72.3, 72.1, 70.9, and 18.0) and another 30 carbons, including eighteen aromatic carbon signals of three benzene rings, two oxygenated methine carbons at *δ*_C_ 89.2 and 88.8, three methyleneoxy carbons at *δ*_C_ 65.1, 64.9 and 62.3, three methoxy carbons at *δ*_C_ 56.9, 56.8 and 56.5, two methine carbons at *δ*_C_ 55.5 and 55.5, two methylene carbons at 35.8 and 32.9.

These spectral features implied that the aglycone of **1** should be a dimer of dihydrobenzofuran neolignan and was the same as vitrifol A [8]. The rhamnose unit was located at C-4 as elucidated by the HMBC correlation between *δ*_H_ 5.33 (H-1′′′) and *δ*_C_ 146.7 (C-4) (Figure 2). The α-orientation of the sugar was determined by the small coupling constant (*J* = 1.5 Hz) of the anomeric proton H-1′′′ and its l-configuration was established by HPLC analysis after acid hydrolysis. By comparison with reported data [9], the relative configuration of H-7/H-8 and H-7′/H-8′ was determined as *threo* according to their coupling constants (*J*_7,8_ = 6.0 Hz and *J*_7′,8′_ = 6.5 Hz). The absolute configuration of **1** was established as 7*R*,8*S* and 7′′*R*,8′′*S* on the basis of the negative Cotton effects at 237 and 290 nm in the circular dichroism (CD) spectrum as shown in Figure S8, Supplementary Materials [10]. From the above analysis, compound **1** was determined to be vitrifol A 4-α-l-rhamnopyranoside, and named as difengpioside A. To the best of our knowledge, this is the first report of a dihydrobenzofuran sesquilignan glucoside from the family Illiciaceae.

Compound **2** was isolated as an amorphous powder. The HRESIMS spectrum showed a peak at *m*/*z* 487.1572 [M + Na]^+^ (calcd. for 487.1575), corresponding to the molecular formula C_23_H_28_O_10_. The IR spectrum displayed the presence of hydroxyl (3412 cm^−1^) and aromatic ring (1595 and 1501 cm^−1^) groups. The ^1^H-NMR spectrum (Table 2) displayed AMX aromatic spin-system signals at *δ*_H_ 6.76 (1H, d, *J* = 8.0 Hz), 6.83 (1H, dd, *J* = 8.0, 1.5 Hz) and 6.97 (1H, d, *J* = 1.5 Hz) , two aromatic H-atom signals at *δ*_H_ 7.02 (1H, s) and 6.56 (1H, s), three aromatic methoxy groups at *δ*_H_ 3.83 (3H, s), 3.82 (3H, s) and 3.79 (3H, s), and an anomeric proton of xylose at *δ*_H_ 4.31 (1H, d, *J* = 7.5 Hz) which indicated a β-configurantion for the xylosyl moiety. The ^13^C-NMR spectrum (Table 2) showed the presence of 23 carbons including twelve aromatic carbon signals of two benzene rings, an oxygenated methine carbon at *δ*_C_ 89.0, a methyleneoxy carbon at *δ*_C_ 72.8, three methoxy carbons at *δ*_C_ 57.8, 56.6 and 56.4, and a methine carbon at *δ*_C_ 52.7. Additionally, five carbon signals (*δ*_C_ 105.0, 77.9, 74.9, 71.2, and 66.9) were ascribed to a xylosyl moiety. The NMR data were quite identical to those of the known compound 2,3-dihydro-7-methoxy-2-(4′-hydroxy-3′-methoxy- phenyl)-3a-*O*-β-d-xylopyranosyloxymethyl-5-benzofuranpropanol [5]. The only difference lies in that the propanol moiety at C-1′ of the known compound was replaced by a methoxyl group in **2**, as confirmed by the HBMC correlation from *δ*_H_ 3.82 (OCH_3_) to *δ*_C_ 151.6 (C-1′) (Figure 2) and the molecular formula of **2**. The xylose unit was located at C-9 as elucidated by the HMBC correlation between *δ*_H_ 4.31 (H-1′′) and *δ*_C_ 72.8 (C-9) and its d-configuration was detected by direct comparison with an authentic sample on HPLC after acid hydrolysis. The absolute configurations of C-7 and C-8 of **2** were determined as 7*R*,8*S*, on the basis of their coupling constant (*J*_7,8_ = 6.5 Hz), indicating H-7 and H-8 to be *threo*, and the negative Cotton effects at 243 and 287 nm in the CD spectrum [10]. Compound **2** was thus identified as (2*R*,3*S*)-2,3-dihydro-5,7-dimethoxy-2-(4′-hydroxy-3′-methoxy- phenyl)-3a-*O*-β-d-xylopyranosyloxymethylbenzofuran and named difengpioside B.

Compound **3** was obtained as an amorphous powder with the molecular formula C_25_H_32_O_10_, as evidenced by HRESIMS (*m*/*z* 515.1920 [M + Na]^+^, calcd. 515.1888). The IR spectrum revealed the presence of hydroxyl (3431 cm^−1^) and aromatic ring (1631 cm^−1^) groups. Five typical carbon signals (*δ*_C_ 105.3, 77.8, 74.9, 71.2, and 66.9) was assigned to a xylose moiety and its β-orientation was determined by the large coupling constant (*J* = 7.5 Hz) of the anomeric proton at *δ*_H_ 4.22. The ^13^C-NMR (Table 2) and HSQC spectra revealed that the remaining signals of **3** contains twelve aromatic carbons of three benzene rings, three methylene carbons including two oxygenated ones at *δ*_C_ 73.9 and 61.6, two methoxy carbons at *δ*_C_ 56.4 and 56.3, three methine carbons at *δ*_C_ 48.1, 47.8 and 37.4. The above data were very similar to those of (+)-isolariciresinol 9-*O*-β-d-xylopyranoside (**5**) [11], except that the difference in the chemical shifts of C-9 and C-9′. This indicated the xylosyl moiety was positioned at C-9′, which was further supported by HMBC correlation observed between H-1′′ (*δ*_H_ 4.22) and C-9′ (*δ*_C_ 73.9). The configuration of the xylosyl unit was established as D by HPLC analysis after acid hydrolysis. The absolute configuration of the chiral centers was established to be the same as that of (+)-isolariciresinol from the results of CD spectral analysis [12]. Therefore, compound **3** was elucidated as (+)-isolariciresinol-9′-*O*-β-d-xylopyranoside, with the trivial name difengpioside C. It is worth noting that although the literature had been reported a compound with the same name as **3** [13], analysis of NMR spectra revealed that the compound actually was identical with (+)-isolariciresinol 9-*O*-β-d-xylopyranoside (**5**).

Compound **4**, isolated as an amorphous powder, had the molecular formula C_32_H_46_O_14_ deduced from HRESIMS *m*/*z* 677.2778 [M + Na]^+^ (calcd. for 677.2780). The IR spectrum revealed the presence of hydroxyl (3426 cm^−1^) and aromatic ring (1631 cm^−1^) groups. The ^1^H-NMR spectrum (Table 2) showed signals of 1,3,4-trisubstituted aromatic ring at *δ*_H_ 6.65 (1H, d, *J* = 8.5 Hz), 6.52 (1H, s) and 6.50 (1H, d, *J* = 8.5 Hz), a methoxy group at *δ*_H_ 3.73 (3H, s), and an anomeric proton of rhamnose at *δ*_H_ 4.63 (1H, d, *J* = 1.5 Hz) which indicated an α–configuration for the sugar moiety. Sixteen carbon signals, including six aromatic carbons, two methylene carbons (including one oxygenated), one methine carbon, one methoxy carbon and six rhamaopyranosyl signals, were supported by the ^13^C-NMR (Table 2) and HSQC spectra. The above spectral data combined with the molecular formula indicated **4** possessed a highly symmetrical skeleton. Acid hydrolysis of **4** liberated the l-rhamnose moiety, which was determined by HPLC analysis. The HMBC correlation between *δ*_H_ 4.63 (H-1′′) and *δ*_C_ 69.4 (C-9) confirmed that L-rhamnose was linked to C-9. The NMR data of **4** were very similar to those of secoisolariciresinol diglucoside [14], except for the sugar moieties, suggesting that **4** was a diphenylbutane-type lignan dirhamnoside. The symmetrical structure feature of **4** indicated its configuration should be either 8S, 8′S or 8R, 8′R. Since two negative Cotton effects at 228 and 280 nm were observed in the CD spectrum [15], the absolute configuration of **4** was determined to be 8*R*,8′*R*. Thus, compound **4** was elucidated as (−)-secoisolariciresinol 9,9′-di-*O*-α-l-rhamnopyranoside, which was named as difengpioside D.

The known compounds were identified as (+)-isolariciresinol 9-*O*-β-d-xylopyranoside (**5**) [11], aviculin (**6**) [16], (+)-isolariciresinol (**7**) [12], (−)-secoisolariciresinol-*O*-α-l-rhamnopyranoside (**8**) [17], 7*S*,8*R*-*threo*-3′,9,9′-trihydroxy-3-methoxy-4′,7-epoxyneolignan-4-*O*-α-l-rhamnopyranoside (**9**) [5], icariside E4 (**10**) [5], *threo*-4,9,9′-trihydroxy-3,3′-dimethoxy-8-*O*-4′-neolignan 7-*O*-α-rhamno-pyranoside (**11**) [18], respectively, by comparison of the spectroscopic data with those reported in the literature.

Inhibitors of NO release are considered as potential anti-inflammatory agents [19]. Since the stem barks of *Illicium difengpi* have been applied for the treatment of rheumatic arthritis in China, the isolated compounds were evaluated for their effects on the inhibition of NO production in LPS-activated RAW264.7 cells. As shown in Table 3, the dihydrobenzofuran-type (**1**, **2**, **9**, and **10**) and aryltetralin-type (**3**, **5**, **6**, and **7**) lignan glycosides exhibited weak inhibitory effect against NO with inhibition ratios in the range of 3.29% to 10.53% at a concentration of 25 µM, while the dibenzylbutane-type lignan glycosides **4** and **8** and neolignan glucoside **11** showed no inhibitory effect at the same concentration.

## 3. Materials and Methods

### 3.1. General Information

Melting points were obtained on an X-4 micro melting point apparatus (Shanghai Jingke Scientific Instrument Co., Ltd, Shanghai, China). Optical rotations were measured with a P-1020 polarimeter (JASCO, Tokyo, Japan). UV spectra were obtained on a UV-2401A spectrophotometer (Shimadzu, Kyoto, Japan). CD spectra were recorded on a J-810 CD spectrometer (JASCO, Tokyo, Japan). IR spectra were measured in a FTS-135 spectrometer (Bio-Rad, Richmond, CA, USA) with KBr pellets. HRESIMS were recorded on a LCMS-IT-TOF spectrometer (Shimadzu, Kyoto, Japan). The NMR spectra were recorded on a DRX-500 spectrometer (Bruker Co., Ettlingen, Germany) with TMS as internal standard, and chemical shifts (δ) were expressed in ppm with reference to the solvent signals. Silica gel (200–300 mesh; Qingdao Marine Chemical Inc., Qingdao, China), D101 macroporous resin (Nankai University, Tianjin, China), ODS (40–63 μm; Merck, Darmstadt, Germany), and Sephadex LH-20 (Amersham Pharmacia Biotech, Uppsala, Sweden) were used for column chromatography. Semipreparative HPLC was performed on an Agilent 1200 apparatus equipped with a UV detector and a Zorbax SB-C-18 (9.4 mm × 25 cm, Agilent Technologies, Santa Clara, CA, USA) column. Analytical HPLC was performed on a Shimadzu HPLC system equipped with a refractive index detector and a CARBOSep COREGEL-87C Ca^+^ (7.8 × 300 mm, 9 µm, Transgenomic Inc., Omaha, NE, USA) column. Fractions were monitored by TLC and spots were visualized by heating silica gel plates sprayed with 10% H_2_SO_4_ in EtOH. Solvents were distilled before use.

### 3.2. Plant Material

The stem barks of *Illicium difengpi* were collected from Longzhou County, Guangxi Province, China, in October 2010 and identified by Prof. H. Tang. A voucher specimen (CTM201002) was deposited at the Guangxi Key Laboratory of Functional Phytochemicals Research and Utilization, Guangxi Institute of Botany, China.

### 3.3. Extraction and Isolation

The air-dried stem barks of *I. difengpi* (14 kg) were extracted with 95% EtOH (20 L × 3) at room temperature for 24 h. The dried EtOH extract (1.3 kg) was suspended in H_2_O and then partitioned successively with petroleum ether (1.5 L × 3), EtOAc (1.5 L × 3) and *n*-BuOH (1.5 L × 3). The *n*-butanol portion (540 g) was applied to a D101 macroporous resin column eluted with water, 10%, 30%, 60%, 90% and 100% methanol. The 30% methanol fraction (32 g) was further purified by an ODS column and a preparative HPLC (CH_3_CN–H_2_O, 15:85) to yield compounds **1** (9 mg) and **4** (12 mg). The 60% methanol fraction (26 g) was subjected to a Sephadex LH-20 column eluted with MeOH to give five fractions (Fr. 1–5). Fr.3 was further separated by an ODS column and preparative HPLC (CH_3_CN–H_2_O, 15:85 to 30:70) to obtain compounds **8** (25 mg), **10** (30 mg) and **11** (8 mg). Fr.4 was subjected to silica gel column chromatography (CHCl_3_–MeOH–H_2_O, 5:1:0.1 to 3:1:0.1) and preparative HPLC (CH_3_CN–H_2_O, 15:85 to 30:70) to obtain compounds **2** (6 mg), **3** (10 mg), **5** (35 mg), **6** (33 mg) and **9** (40 mg). The 90% methanol fraction (12 g) was further purified by silica gel column chromatography (CHCl_3_–MeOH, 5:1 to 0:1) and preparative HPLC (CH_3_CN–H_2_O, 30:70) to yield compound **7** (10 mg).

### 3.4. Compound Characterization

Difengpioside A (**1**): Amorphous powder; ${\left[\alpha \right]}_{\text{D}}^{20}$ +25 (*c* 0.2, MeOH); IR (KBr) ν_max_: 3406, 2933, 1609, 1511, 1267, 1030 cm^−1^; UV (MeOH) *λ*max (logε) nm: 204 (4.47), 281 (3.55); CD (MeOH, nm) *λ*_max_ (∆ε) 290 (−3.18), 254 (−2.55), 237 (−3.27); ^1^H and ^13^C-NMR data, see Table 1; HRESIMS *m*/*z* 707.2581 [M + Na]^+^ (calcd for C_36_H_44_O_13_Na, 707.2674).

Difengpioside B (**2**): white amorphous powder; ${\left[\alpha \right]}_{\text{D}}^{20}$ +21 (*c* 0.2, MeOH); IR (KBr) ν_max_: 3412, 2937, 1595, 1501, 1461, 1124, 1034 cm^−1^; UV (MeOH) *λ*max (logε) nm: 206 (4.87), 281 (3.58); CD (MeOH, nm) *λ*_max_ (∆ε) 287 (−3.57), 254 (−3.39), 243 (−4.10), 213 (−2.90); ^1^H and ^13^C-NMR data, see Table 2; HRESIMS *m*/*z* 487.1572 [M + Na]^+^ (calcd for C_23_H_28_O_10_Na, 487.1575).

Difengpioside C (**3**): white amorphous powder; ${\left[\alpha \right]}_{\text{D}}^{20}$ +16 (*c* 0.2, MeOH); IR (KBr) ν_max_: 3431, 2924, 1631, 1384, 1049 cm^−1^; UV (MeOH) *λ*_max_ (logε) nm: 204 (3.45), 281 (2.59); CD (MeOH, nm) *λ*_max_ (∆ε) 292 (−0.21), 277 (0.16), 239 (0.20), 213 (0.56); ^1^H and ^13^C-NMR data, see Table 2; HRESIMS *m/z* 515.1967 [M + Na]^+^ (calcd for C_25_H_32_O_10_Na,515.1888).

Difengpioside D (**4**): white amorphous powder; ${\left[\alpha \right]}_{\text{D}}^{20}$ −32 (*c* 0.2, MeOH); IR (KBr) ν_max_: 3426, 2932, 1631, 1141 cm^−1^; UV (MeOH) *λ*_max_ (logε) nm: 204 (4.68), 281 (3.64); CD (MeOH, nm) *λ*_max_ (∆ε) 280 (−0.89), 228 (−1.98), 211 (−5.35); ^1^H- and ^13^C-NMR data, see Table 2; HRESIMS *m*/*z* 677.2778 [M + Na]^+^ (calcd for C_32_H_46_O_14_Na, 677.2780).

### 3.5. Determination of the Absolute Configuration of the Sugars in Compounds ***1**−**4***

Each compound (1.5 mg) was dissolved in 0.5 M HCl (0.2 mL) and heated at 80 °C for 2 h. The mixture was desalinated by passing through columns of anion and cation exchange resin and then filtered. The filtrate was directly analyzed by a Shimadzu HPLC system equipped with a refractive index detector and a CARBOSep COREGEL-87C Ca^+^ column (7.8 × 300 mm, 9 µm, Transgenomic Inc.) at 85 °C with elution of HPLC grade water for 18 min at a flow rate of 0.5 mL/min. The injection volume was 20 µL. The standards l-rhamnose and d-xylose were treated by the same water and chromatographic conditions. The sugars from each compound were identified by comparison of their retention times with those for authentic standards (*t*_R_: 13.67 min for l-rhamnose, 13.13 min for d-xylose).

### 3.6. NO Production Inhibition Assay

Assays for NO production were carried out as previously described [20]. Briefly, RAW 264.7 macrophages were harvested and seeded in 96-well plates (3 × 10^4^ cells/well) for measurement of NO production. The plates were pretreated with various samples for 30 min and then incubated with 1 μg/mL LPS for 24 h. The inhibitory effects of the isolated compounds on NO production were evaluated by using the Griess reagent.

## 4. Conclusions

Eleven compounds, including four new lignan glucosides **1**–**4**, named difengpiosides A–D, were isolated from the *n*-BuOH-soluble fraction of an EtOH extract of the stem barks of *I. difengpi*. All compounds showed weak or no inhibitory activities against NO production at the concentration of 25 µM, indicating that the polar constituents of this plant showed no inflammatory activity *in vitro*.

## Figures and Tables

**Table 1:** NMR data of compound **1** (in CD_3_OD, *J* in Hz).

No.	δ_C_	δ_H_	No.	δ_C_	δ_H_
1	138.6		1′′	137.1	
2	111.4	7.01 d (2.0)	2′′	112.0	6.72 s
3	152.2		3′′	145.3	
4	146.7		4′′	147.5	
5	119.7	7.07 d (8.5)	5′′	129.9	
6	119.2	6.89 dd (8.5, 2.0)	6′′	117.9	6.72 s
7	88.8	5.58 d (6.0)	7′′	32.9	2.61 t (7.5)
8	55.5	3.49 m	8′′	35.8	1.80 m
9	65.1	3.83 m	9′′	62.3	3.55 t (6.5)
1′	137.0		1′′′	101.5	5.33 d (1.5)
2′	112.0	6.92 s	2′′′	72.1	4.04 dd (3.5, 2.0)
3′	145.6		3′′′	72.3	3.86 m
4′	149.3		4′′′	73.9	3.43 t (9.5)
5′	130.1		5′′′	70.9	3.78 m
6′	115.8	6.92 s	6′′′	18.0	1.20 d (6.0)
7′	89.2	5.52 d (6.5)	3-MeO	56.5	3.80 s
8′	55.5	3.49 m	3′-MeO	56.8	3.85 s
9′	64.9	3.75 m	3′′-MeO	56.9	3.85 s

**Table 2:** NMR data of compounds **2**–**4** (in CD_3_OD, *J* in Hz).

No.	2		3		4	
	δ_C_	δ_H_	δ_C_	δ_H_	δ_C_	δ_H_
1	134.7		138.6		133.4	
2	110.6	6.97 d (1.5)	113.9	6.67 d (2.0)	113.3	6.52 s
3	149.0		148.9		148.9	
4	147.4		145.9		145.6	
5	116.1	6.76 d (8.0)	116.0	6.72 d (8.0)	115.9	6.65 d (8.5)
6	119.7	6.83 dd (8.0, 1.5)	123.2	6.59 dd (8.0, 2.0)	122.7	6.50 d (8.5)
7	89.0	5.44 d (6.5)	47.8	3.84 br d (11.5)	36.2	2.62 d (7.5)
8	52.7	3.57 dd (13.5, 6.5)	48.1	1.75 m	41.6	2.05 m
9	72.8	3.99 dd (9.5, 7.5) 3.81 overlap	61.6	3.35 dd (11.5, 3.0) 3.68 dd (11.5, 3.0)	69.4	3.34 dd (9.5, 5.5) 3.80 dd (9.5, 6.5)
1′	151.6		128.9			
2′	96.1	6.56 s	112.4	6.63 s		
3′	144.9		147.2			
4′	155.6		145.2			
5′	119.0		117.4	6.17 s		
6′	111.6	7.02 s	134.1			
7′			33.7	2.81 br d (7.5)		
8′			37.4	2.13 m		
9′			73.9	3.63 dd (10.0, 6.0) 3.90 dd (10.0, 6.0)		
1′′	105.0	4.31 d (7.5)	105.3	4.22 d (7.5)	102.3	4.63 d (1.5)
2′′	74.9	4.04 dd (8.5, 7.5)	74.9	3.19 m	72.4	3.81 dd (3.5, 1.5)
3′′	77.9	3.86 dd (9.0, 4.0)	77.8	3.30 m	72.6	3.67 dd (9.5, 3.5)
4′′	71.2	3.50 m	71.2	3.48 m	73.9	3.36 t (9.5)
5′′	66.9	3.87 dd (11.0, 5.0) 3.20 d (11.0)	66.9	3.20 m 3.85 br d (11.5)	70.1	3.63 dd (9.5, 6.0)
6′′					18.0	1.25 d (6.0)
3-MeO	56.4	3.83 s	56.3	3.77 s	56.3	3.73 s
1′-MeO	56.6	3.82 s				
3′-MeO	57.8	3.79 s	56.4	3.79 s	56.3	3.73 s

**Table 3:** Inhibitory effects of compounds **1**–**11** on NO production.

Compound	Conc. (μM)	Inhibitory Rate (%)
**1**	25	7.24
**2**	25	5.69
**3**	25	4.61
**4**	25	−0.65
**5**	25	10.53
**6**	25	3.95
**7**	25	3.29
**8**	25	1.32
**9**	25	3.30
**10**	25	4.05
**11**	25	0.66
MG132 ^a^	0.125	91.19

^a^ Positive control, carbobenzoxy-leu-leu-leucinal.

## Supplementary Materials

Supplementary materials can be accessed at: http://www.mdpi.com/1420-3049/21/5/607/s1. HRESIMS, 1D- and 2D-NMR, IR, and CD spectra of four new compounds **1**–**4**.
