# Supplementary material for: Lignan Glucosides from the Stem Barks of Illicium difengpi
Source: Molecules. 2016 May 10;21(5):607. doi: 10.3390/molecules21050607 (PMC6273417; doi:10.3390/molecules21050607)
Supplement: Supplementary file 1 [file molecules-21-00607-s001.pdf]

Supplementary Materials: Lignan Glucosides from the Stem Barks of *Illicium difengpi*

Zheng-Hong Pan, De-Sheng Ning, Si-Si Huang, Ling Cheng, Meng-Wen Xia, Li-Yan Peng and Dian-Peng Li

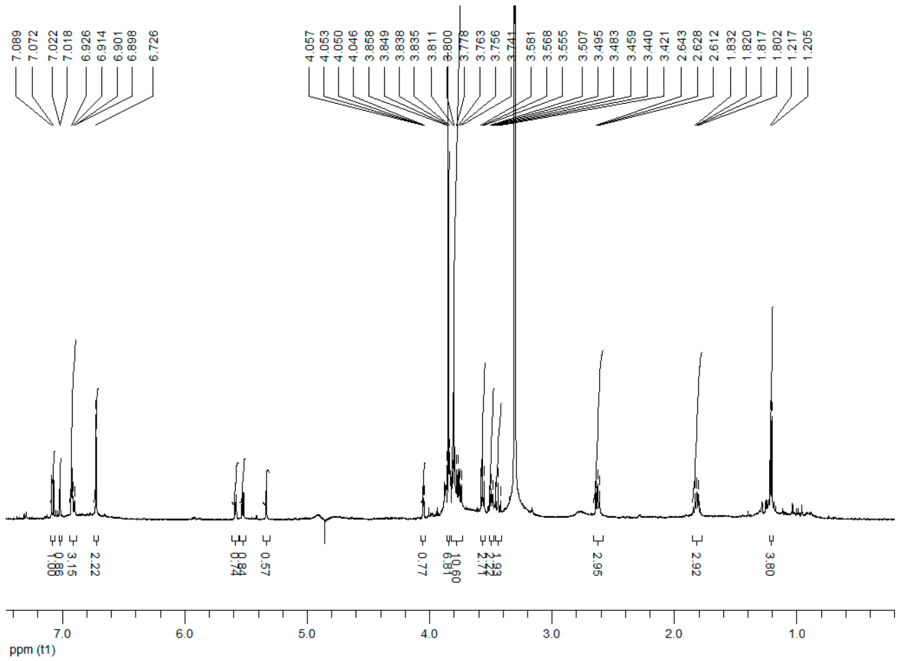

Figure S1. ^{1}H-NMR spectrum of difengpioside A (**1**) in CD_3OD (500 MHz).

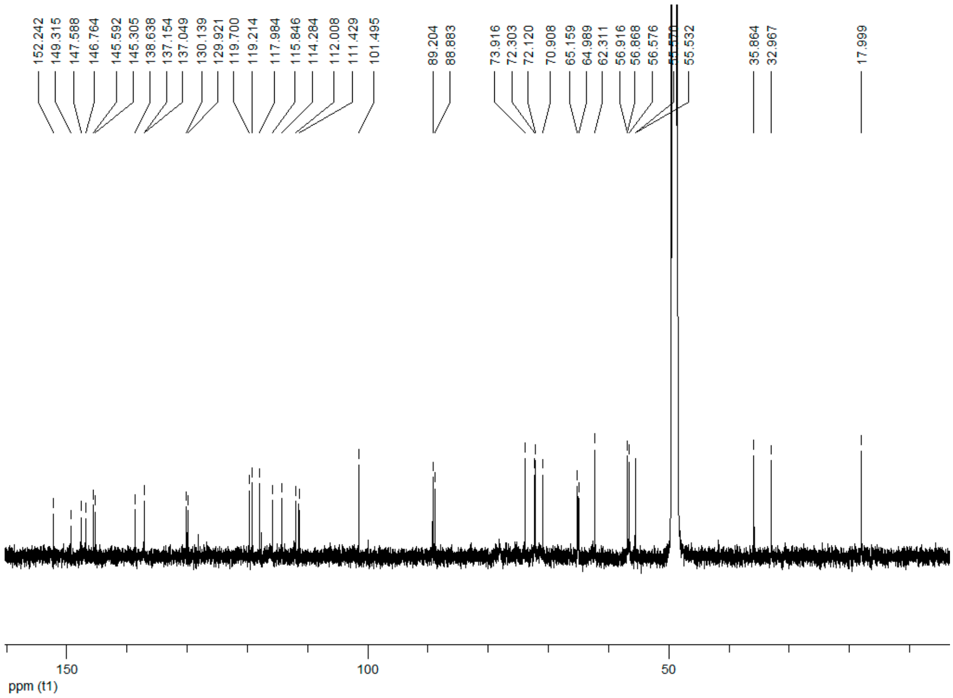

Figure S2. ^{13}C-NMR spectrum of difengpioside A (**1**) in CD_3OD (125 MHz).

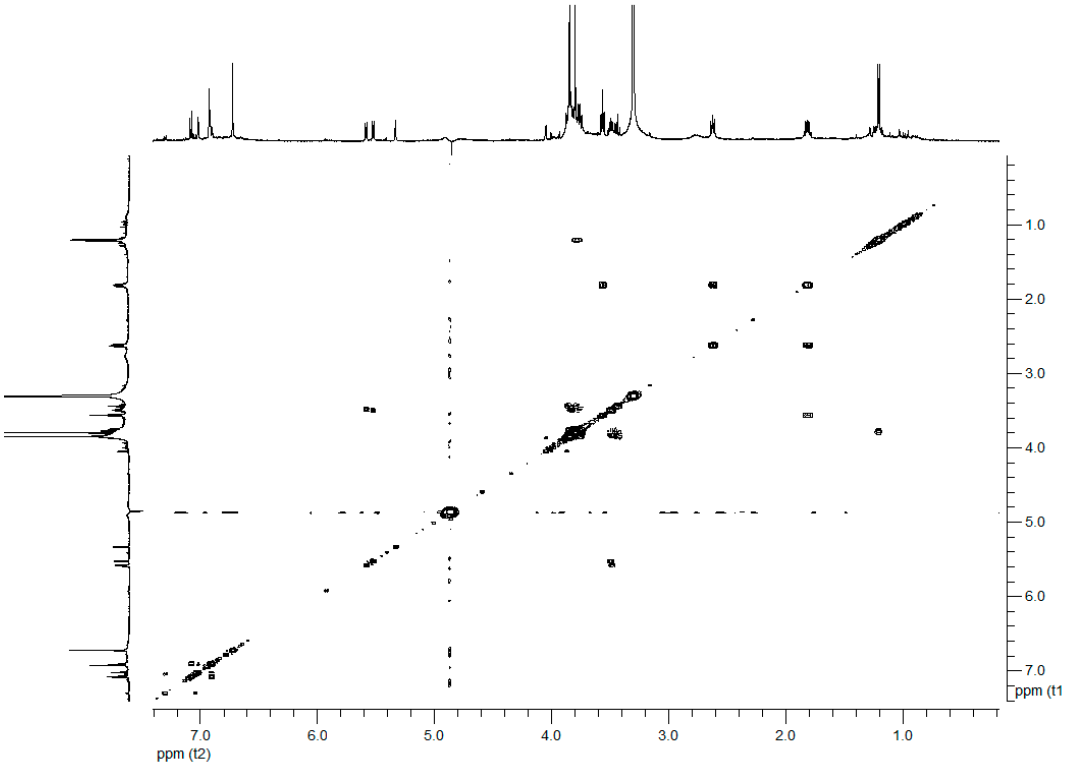

Figure S3. ^{1}H-^{1}H COSY spectrum of difengpioside A (**1**).

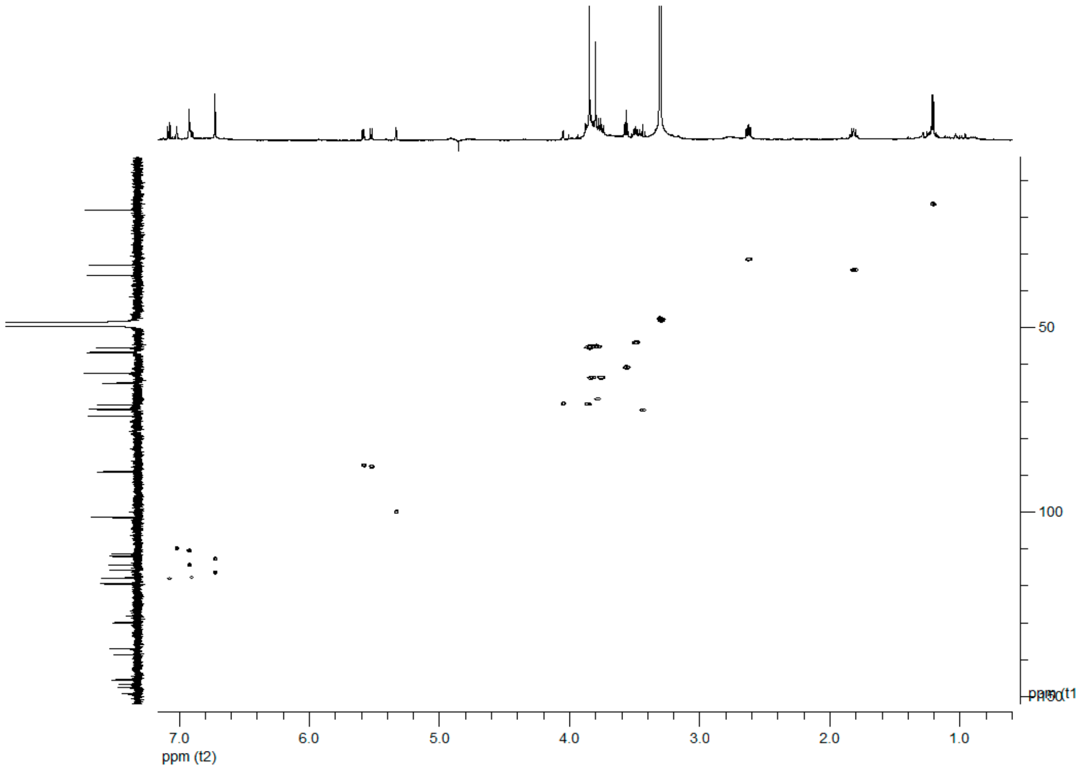

Figure S4. HSQC spectrum of difengpioside A (**1**).

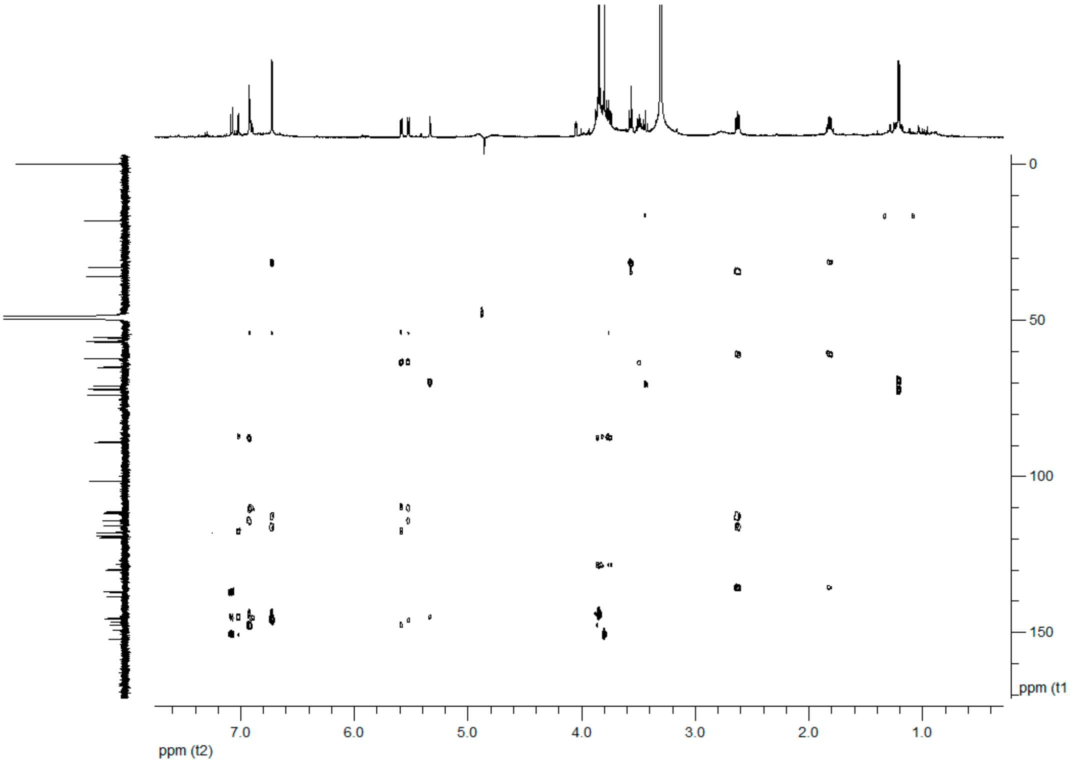

Figure S5. HMBC spectrum of difengpioside A (**1**).

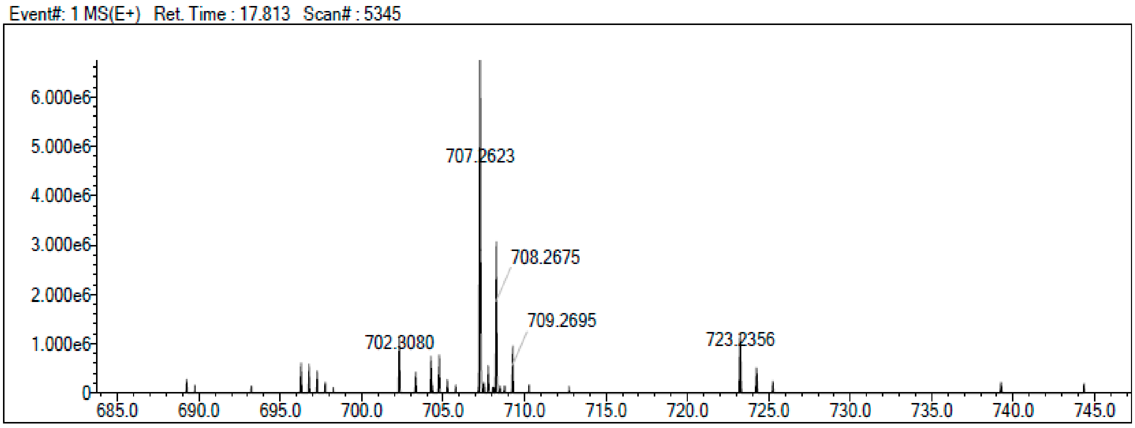

Figure S6. Positive HRESIMS spectrum of difengpioside A (**1**).

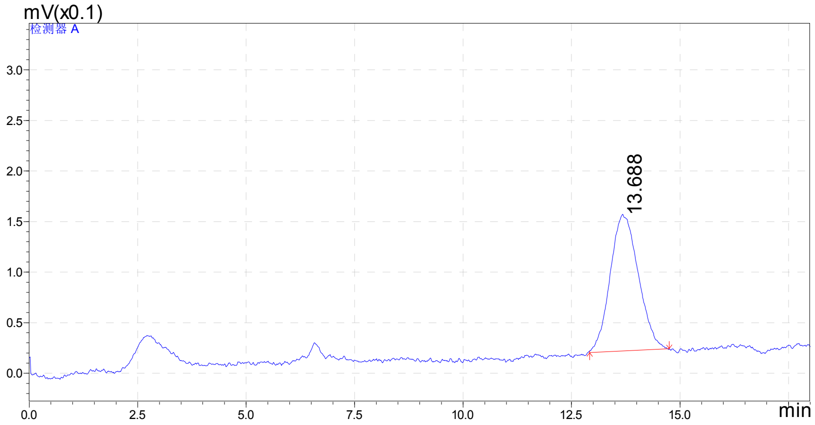

Figure S7. HPLC Analysis of the sugar of difengpioside A (**1**). Top: L-rhamnose; Below: sugar of compound **1**.

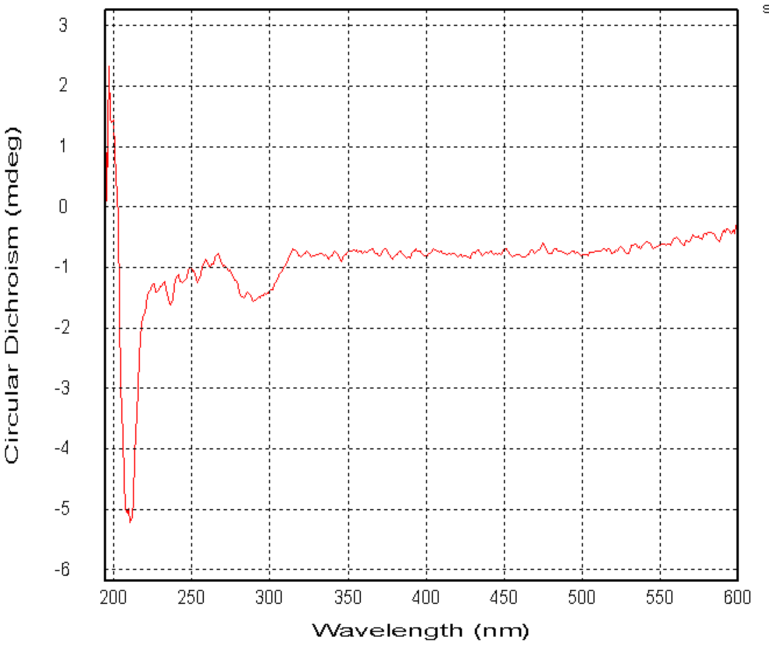

Figure S8. CD Spectrum of difengpioside A (**1**) in MeOH.

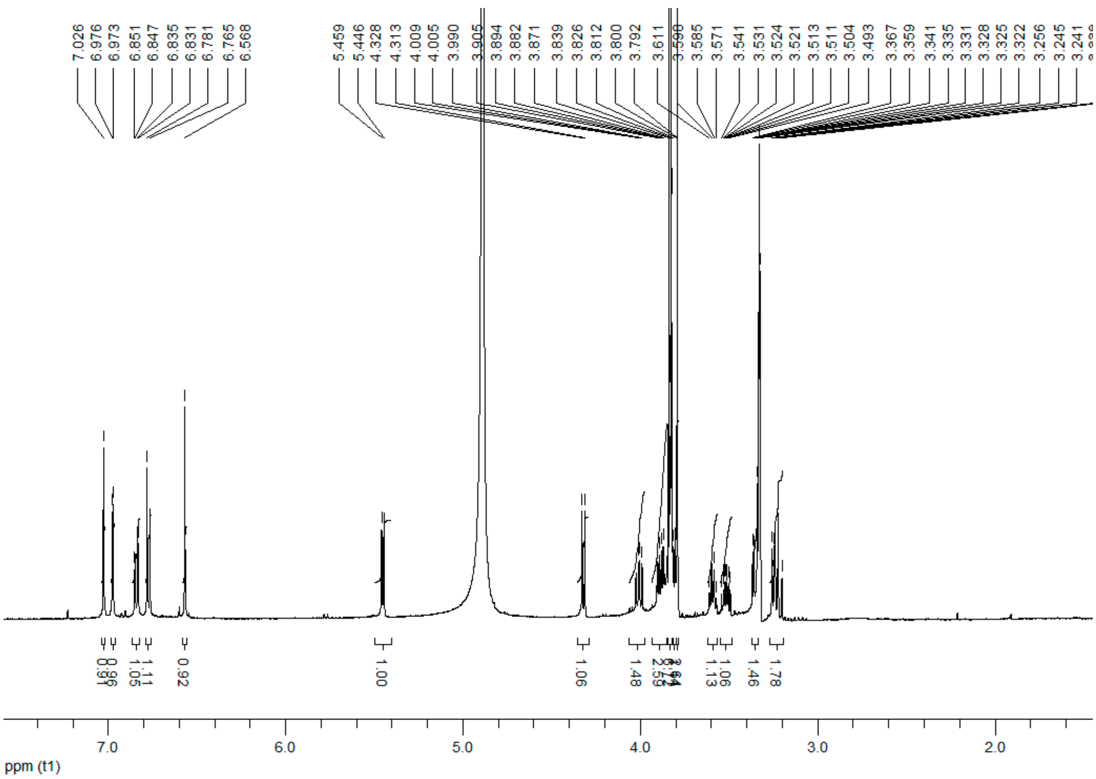

Figure S9. ^{1}H-NMR spectrum of difengpioside B (**2**) in CD_3OD (500 MHz).

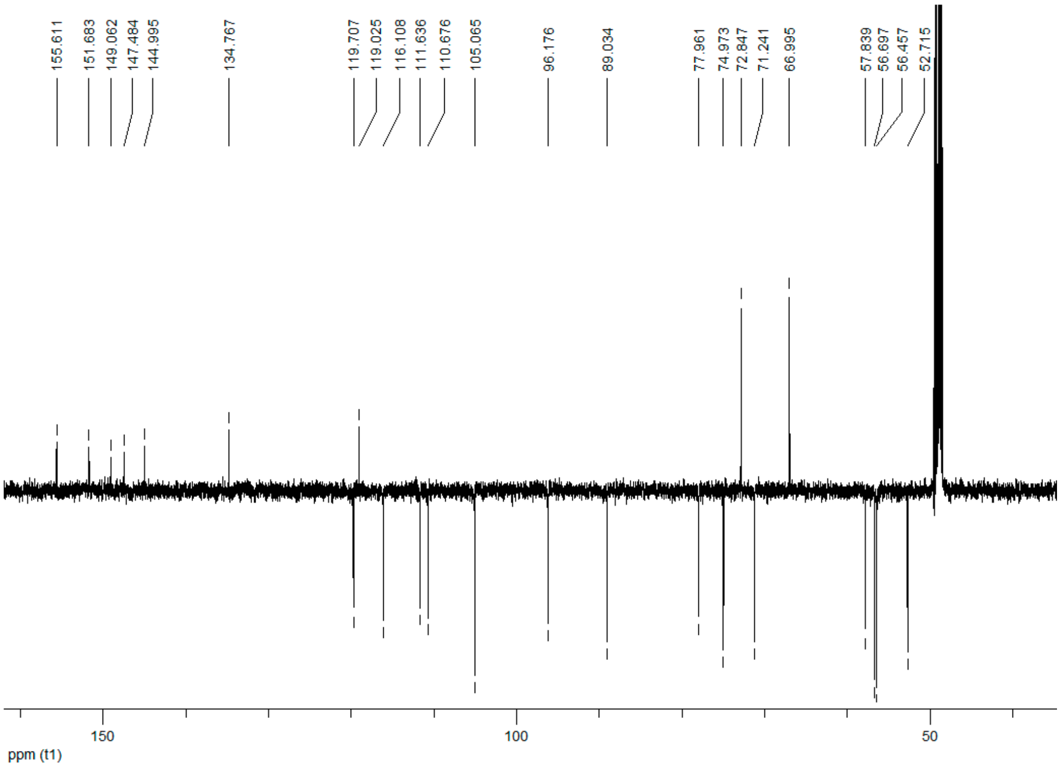

Figure S10. DEPTQ spectrum of difengpioside B (**2**) in CD_3OD (125 MHz).

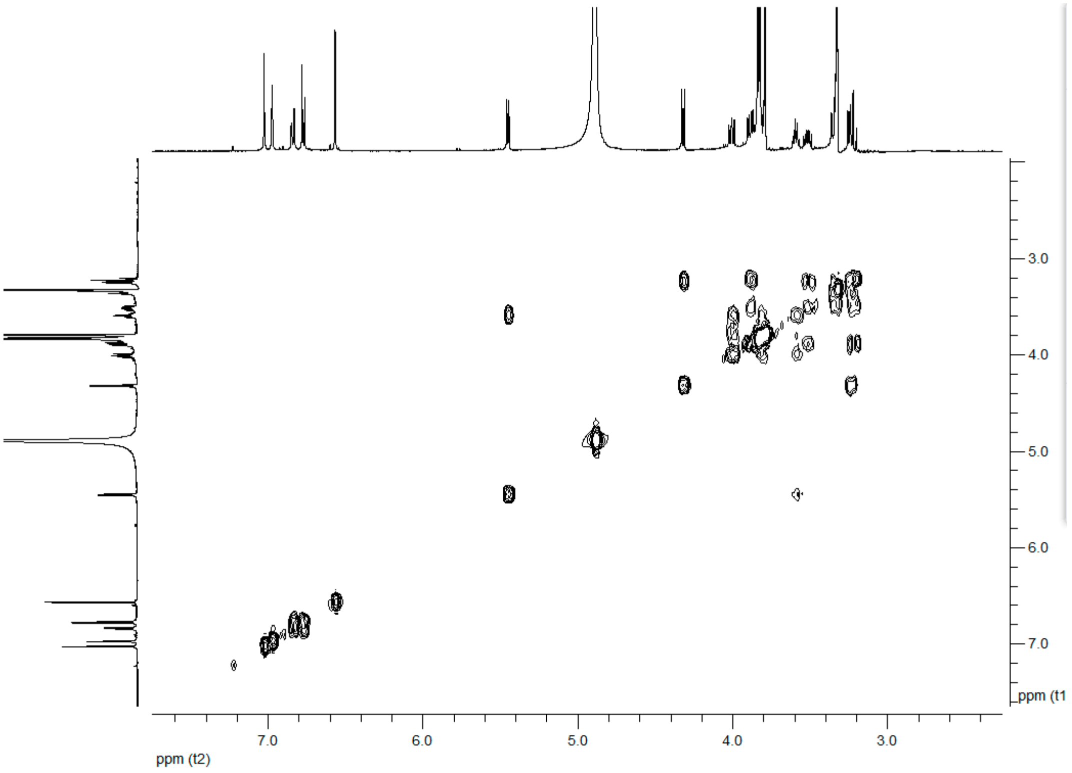

Figure S11. ^{1}H-^{1}H COSY spectrum of difengpioside B (**2**).

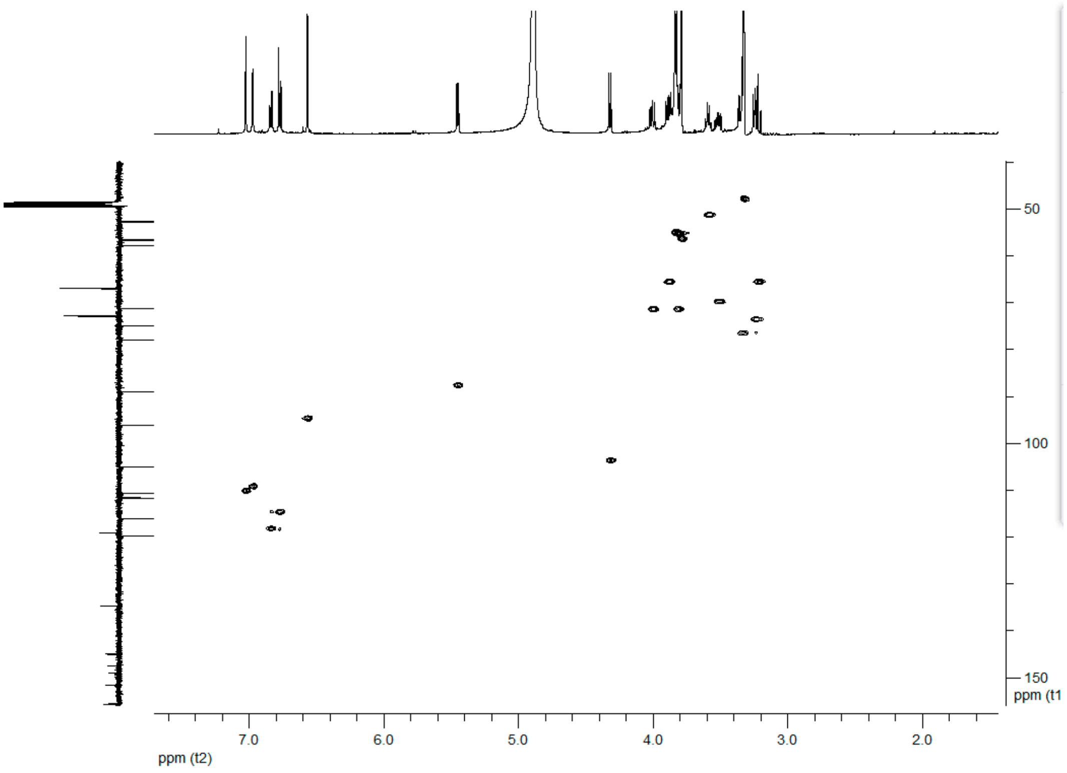

Figure S12. HSQC spectrum of difengpioside B (**2**).

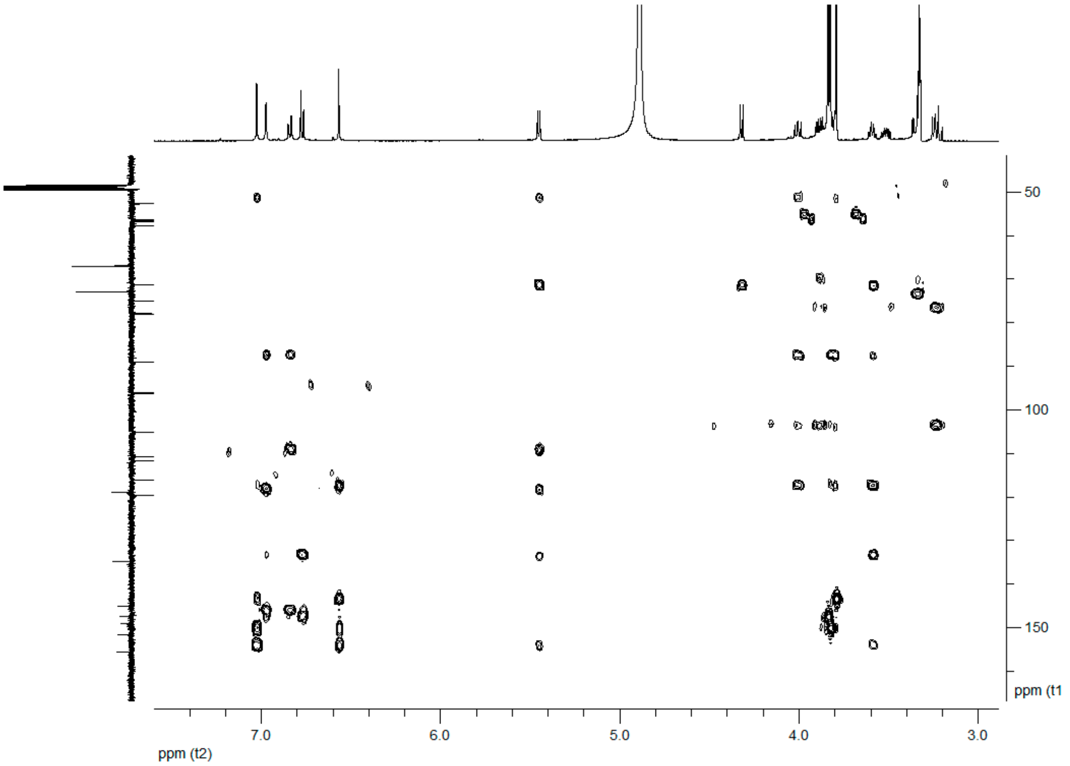

Figure S13. HMBC spectrum of difengpioside B (**2**).

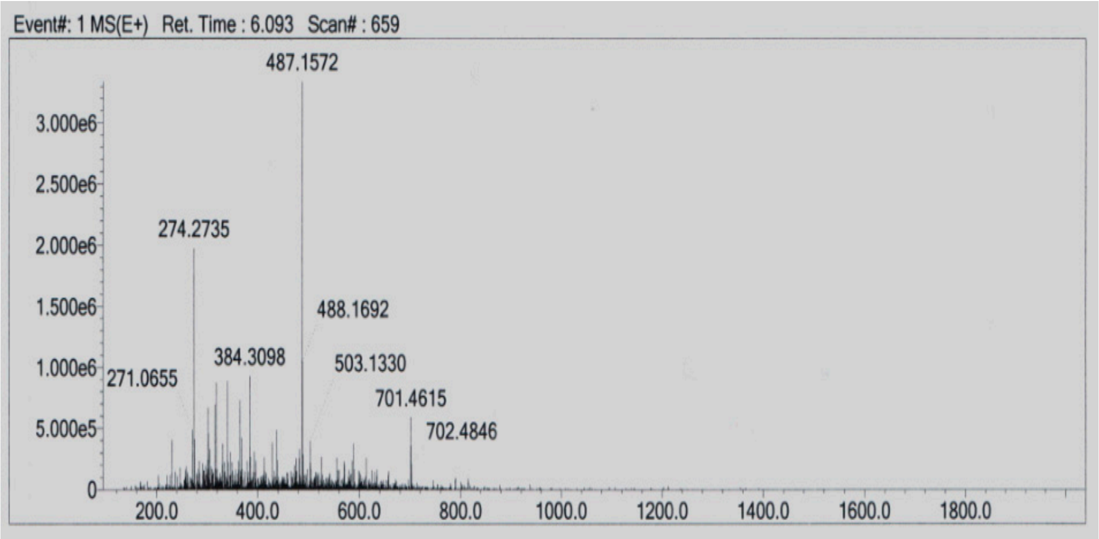

Figure S14. Positive HRESIMS spectrum of difengpioside B (**2**).

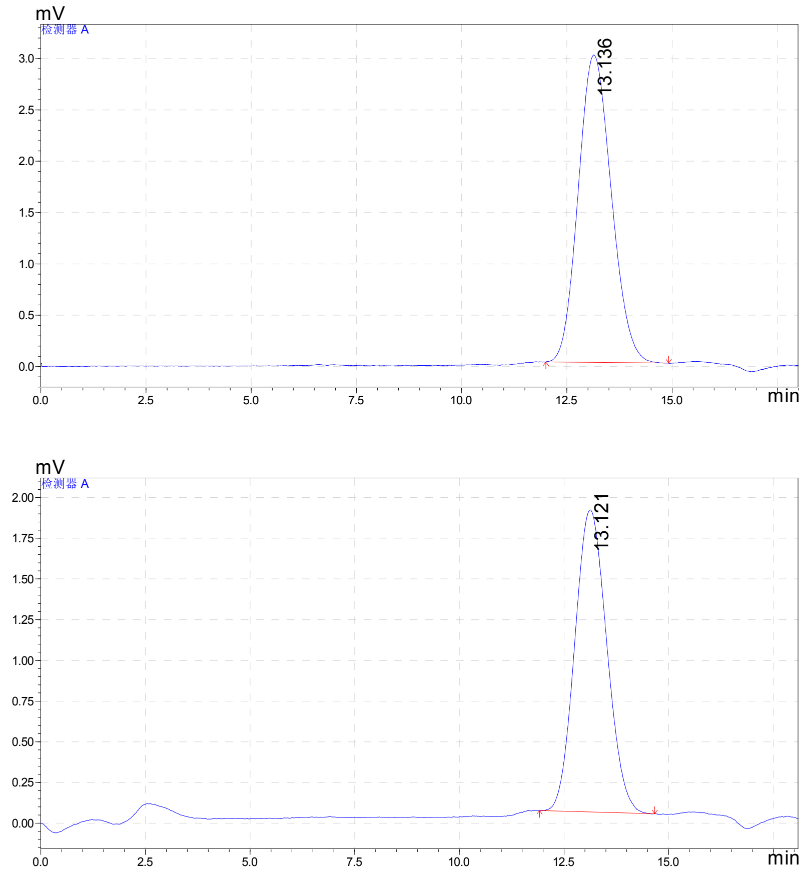

Figure S15. HPLC Analysis of the sugar of difengpioside B (**2**). **Top**: D-xylose; **Below**: sugar of compound **2**.

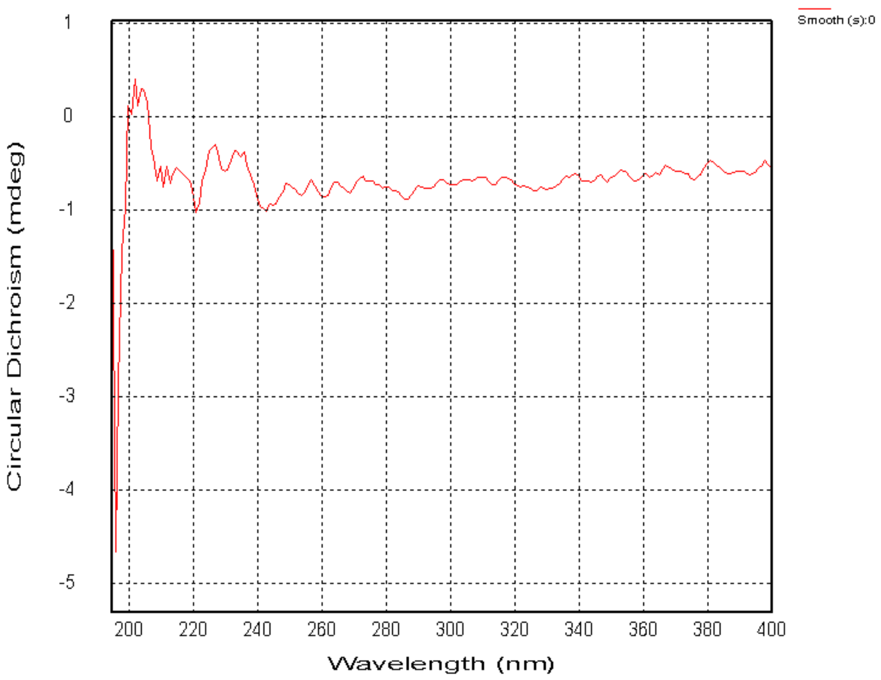

Figure S16. CD Spectrum of difengpioside B (**2**) in MeOH.

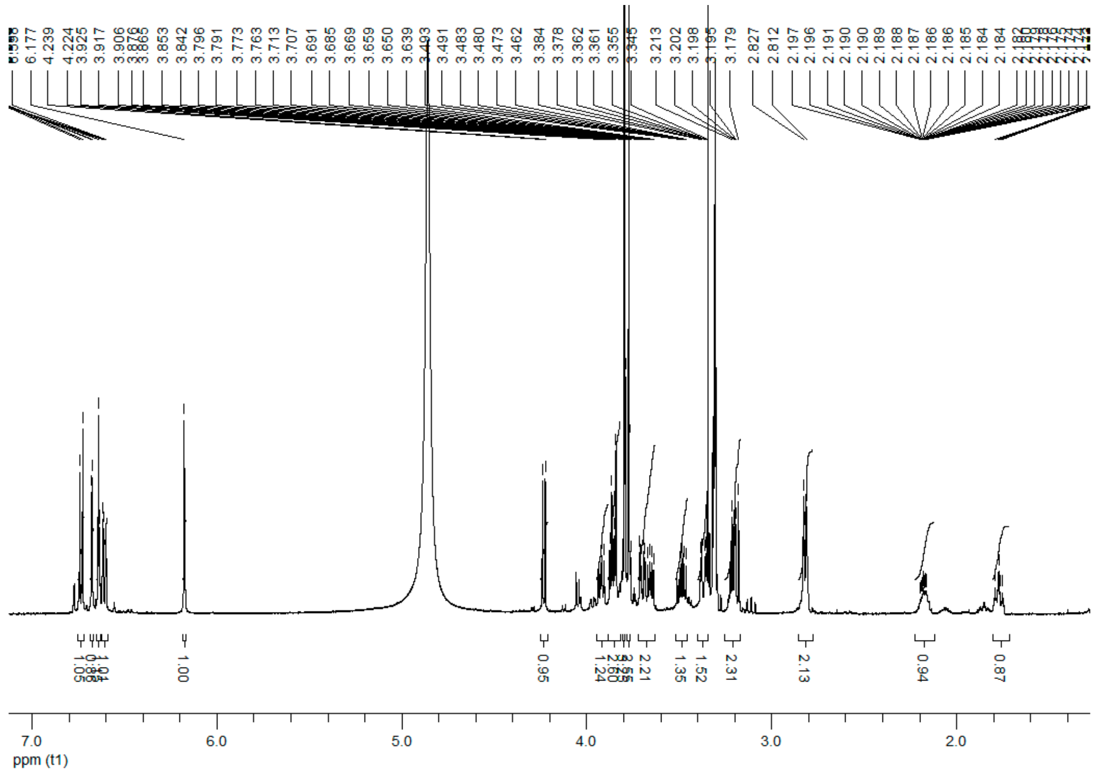

Figure S17. ^{1}H-NMR Spectrum of difengpioside C (**3**) in CD_3OD (500 MHz).

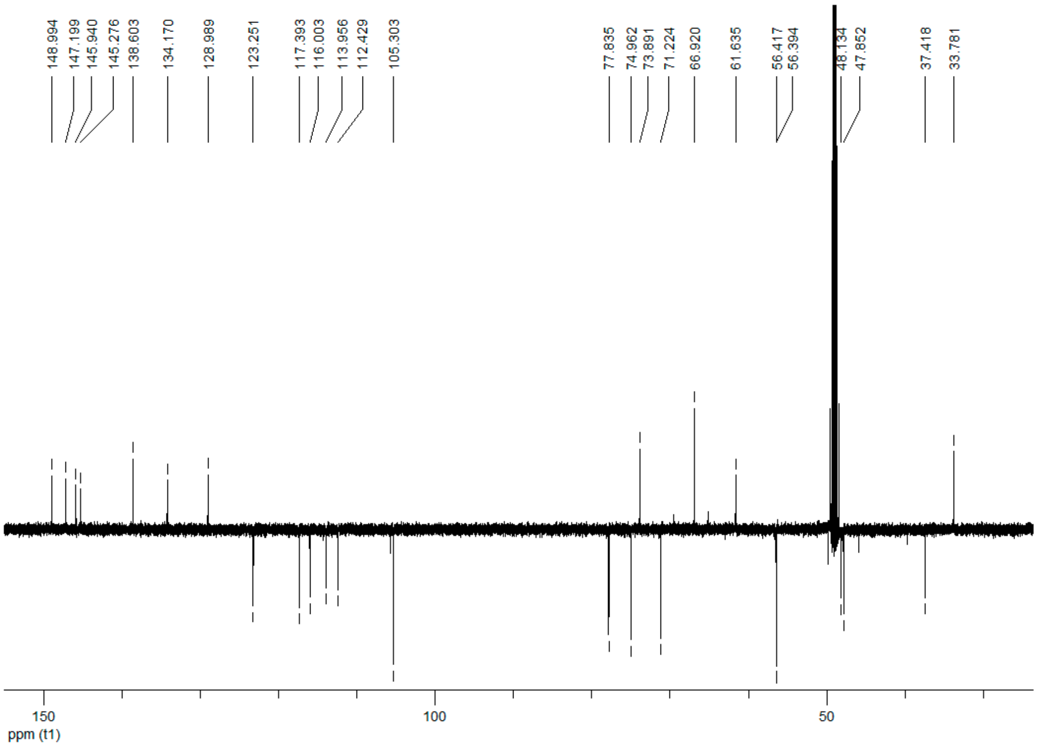

Figure S18. ^{13}C-NMR Spectrum of difengpioside C (**3**) in CD_3OD (125 MHz).

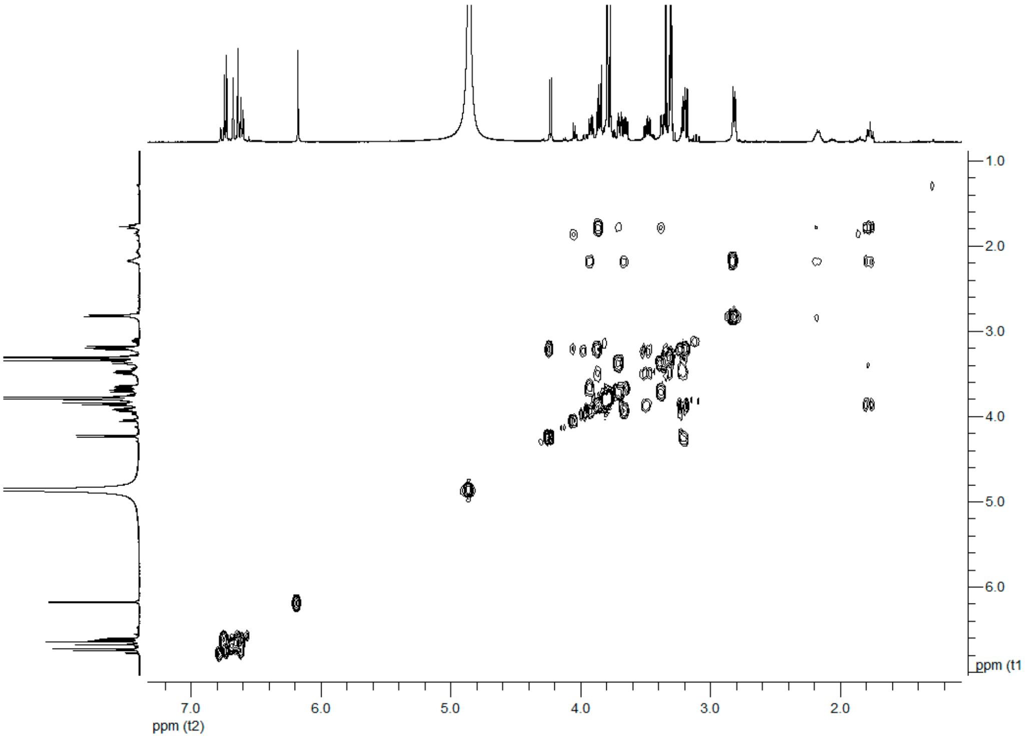

Figure S19. ^{1}H-^{1}H COSY Spectrum of difengpioside C (**3**).

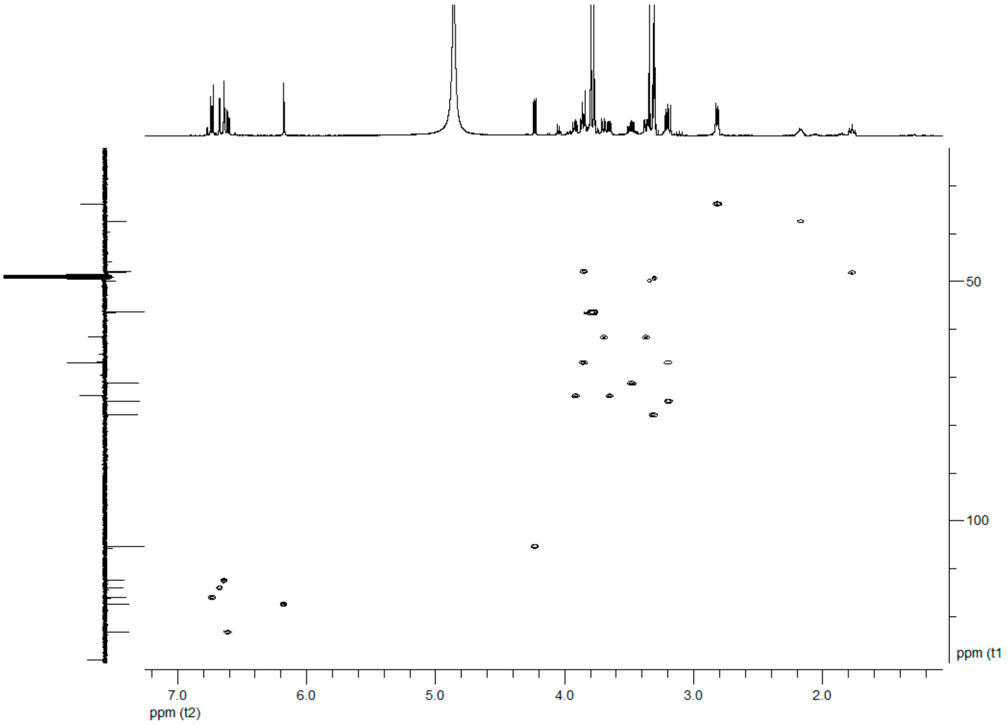

Figure S20. HSQC Spectrum of difengpioside C (**3**).

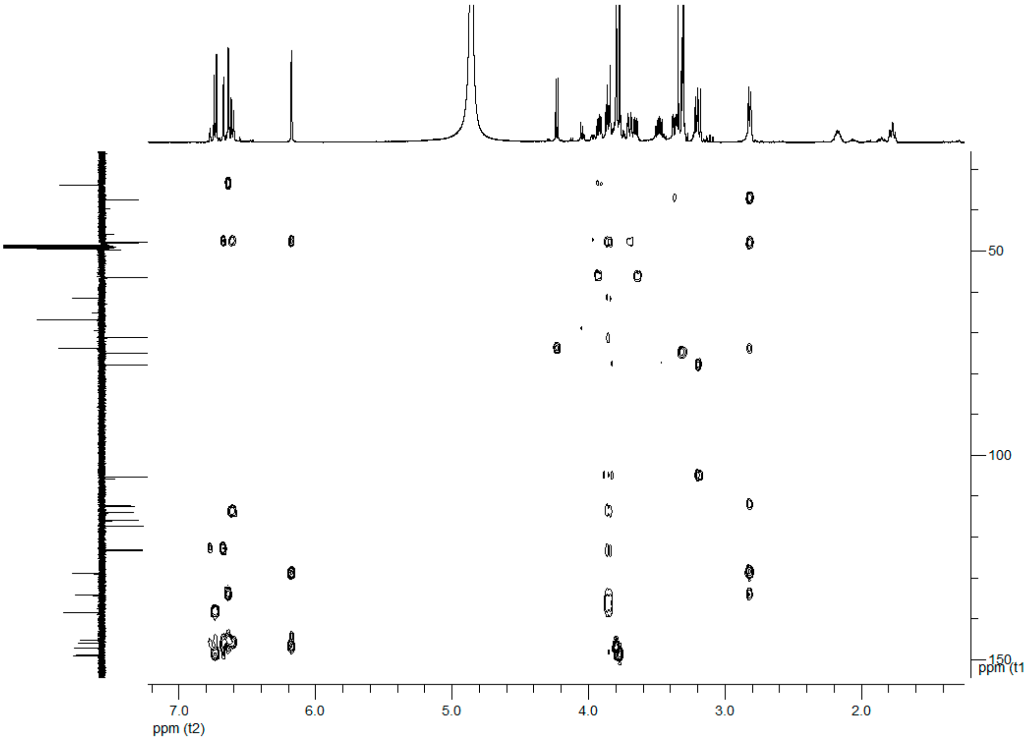

Figure S21. HMBC Spectrum of difengpioside C (**3**).

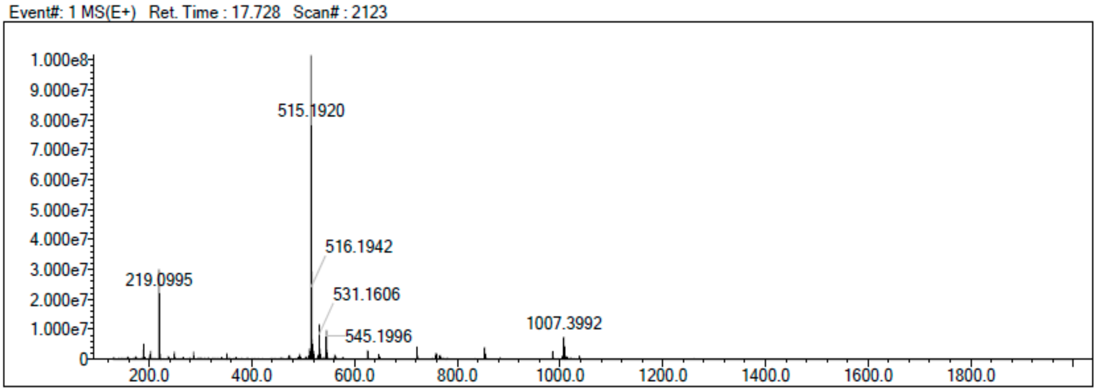

Figure S22. Positive HRESIMS spectrum of difengpioside C (**3**).

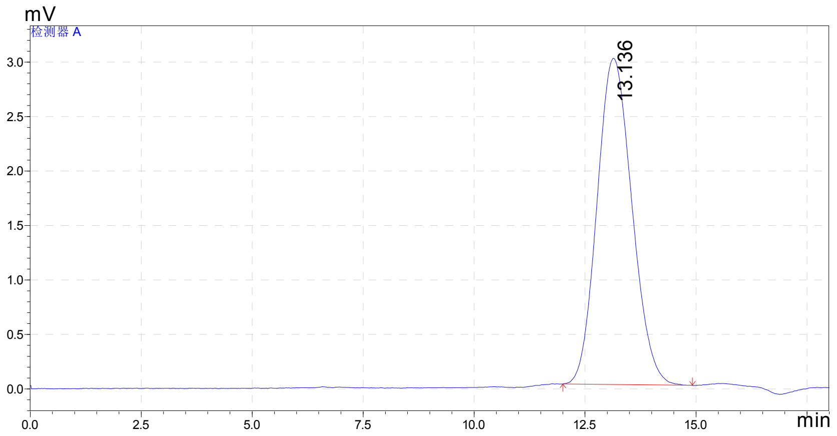

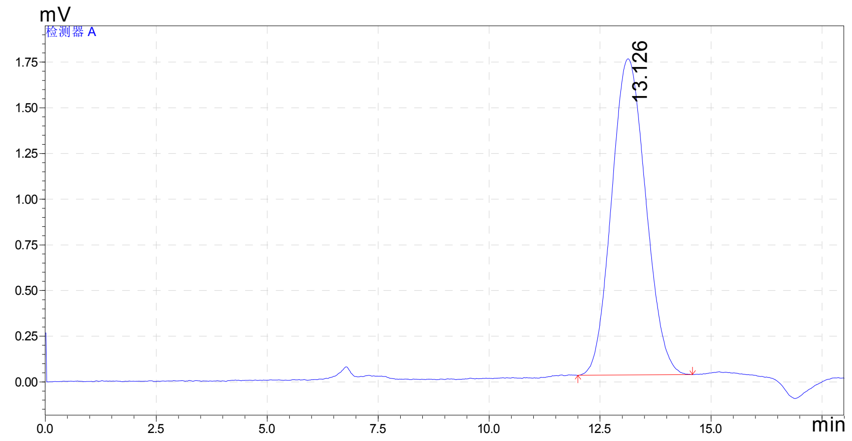

Figure S23. HPLC Analysis of the sugar of difengpioside C (**3**). Top: D-xylose; Below: sugar of compound **3**.

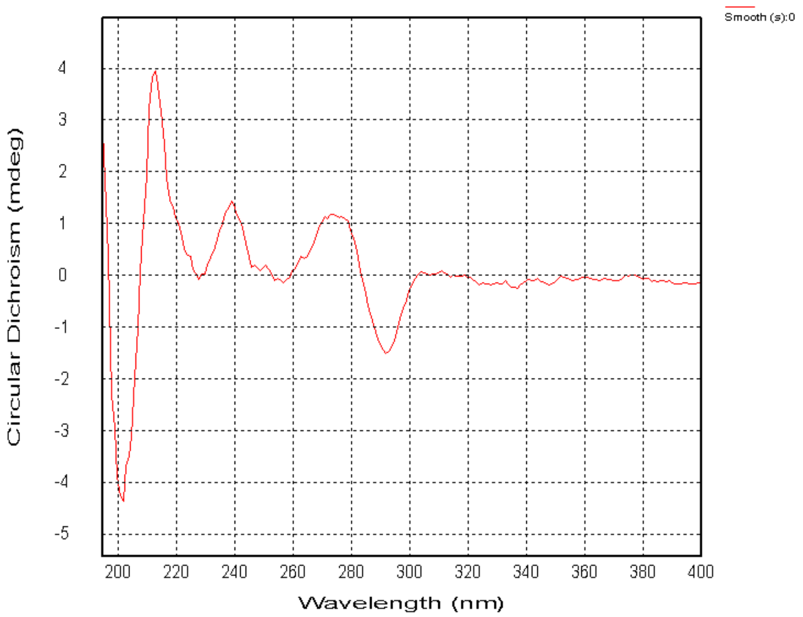

Figure S24. CD Spectrum of difengpioside C (**3**) in MeOH.

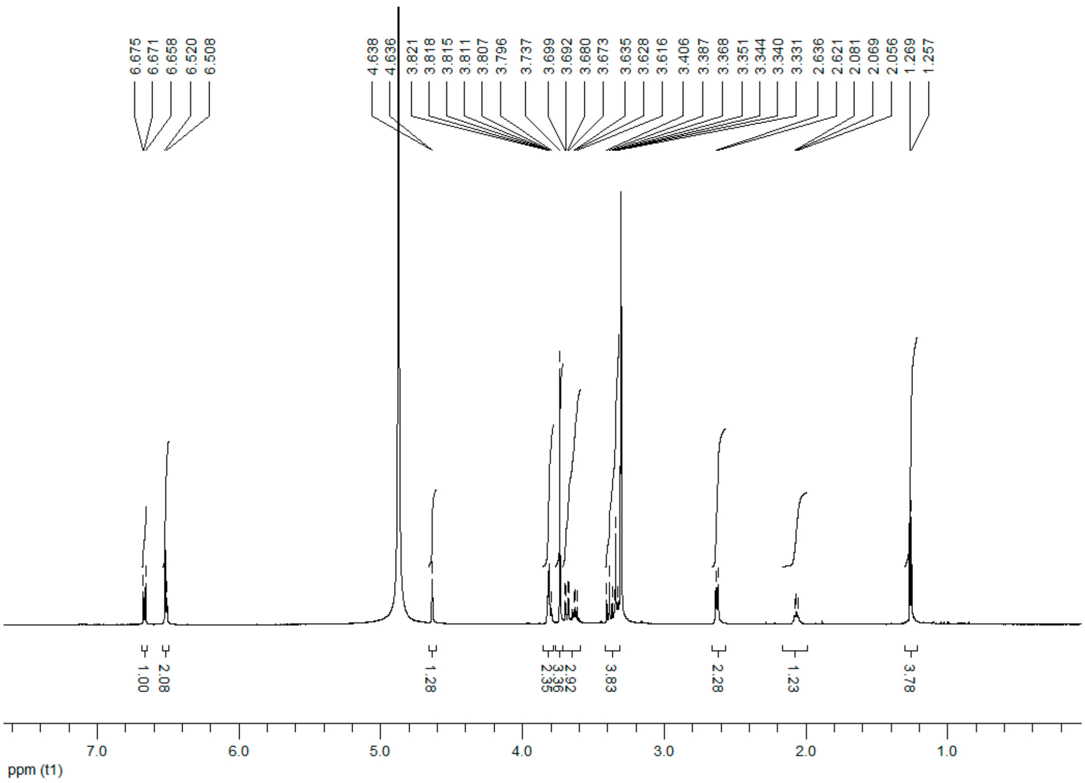

Figure S25. ^{1}H-NMR Spectrum of difengpioside D (**4**) in CD_3OD (500 MHz).

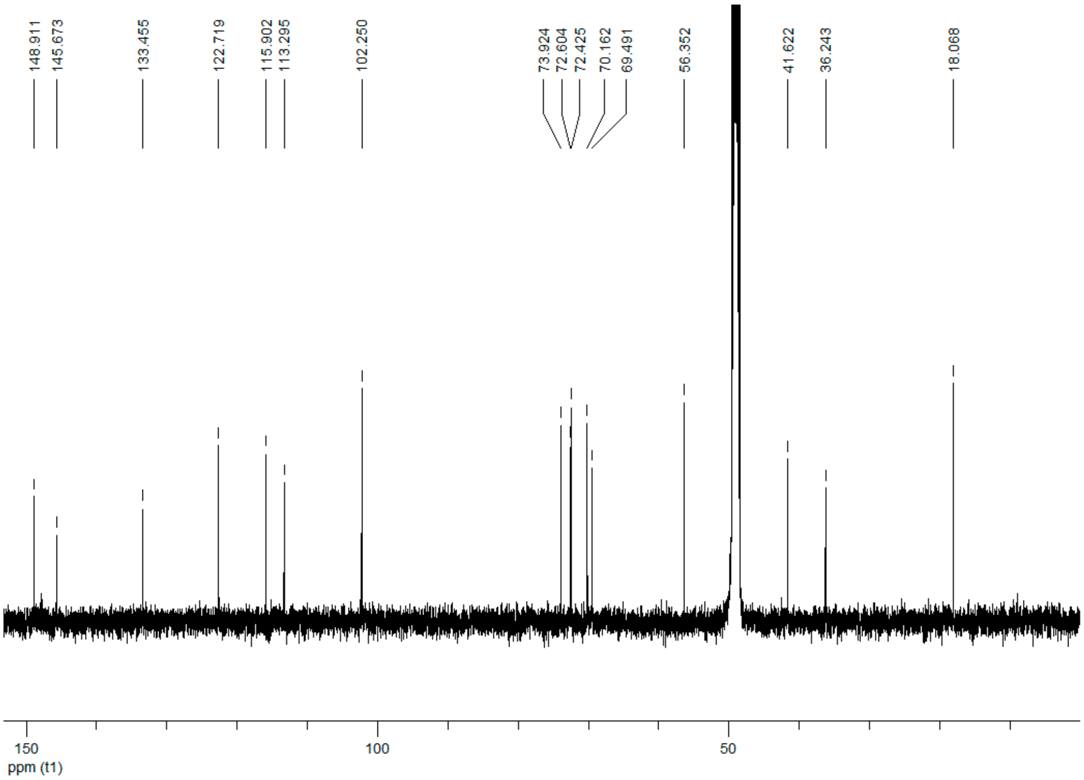

Figure S26. ^{13}C-NMR Spectrum of difengpioside D (**4**) in CD_3OD (125 MHz).

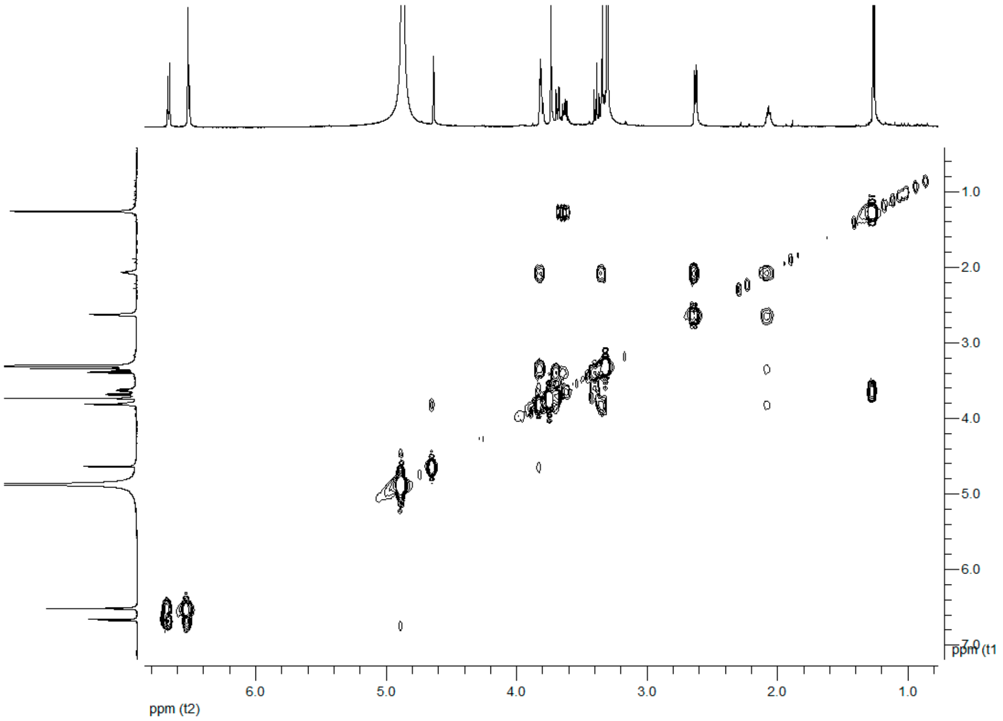

Figure S27. ^{1}H-^{1}H COSY Spectrum of difengpioside D (**4**).

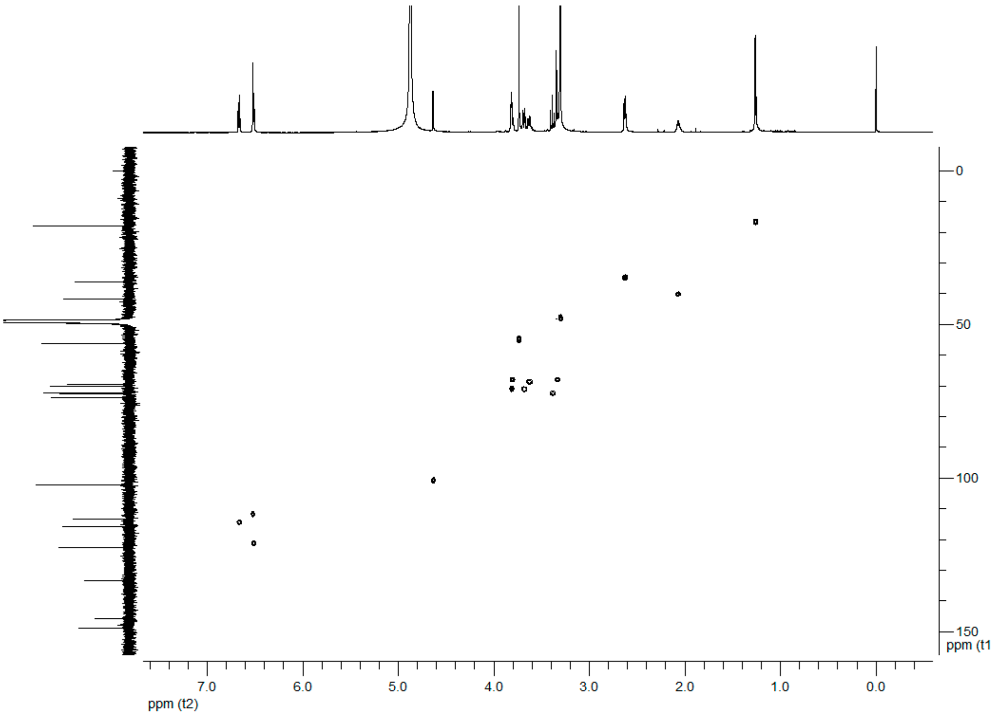

Figure S28. HSQC Spectrum of difengpioside D (**4**).

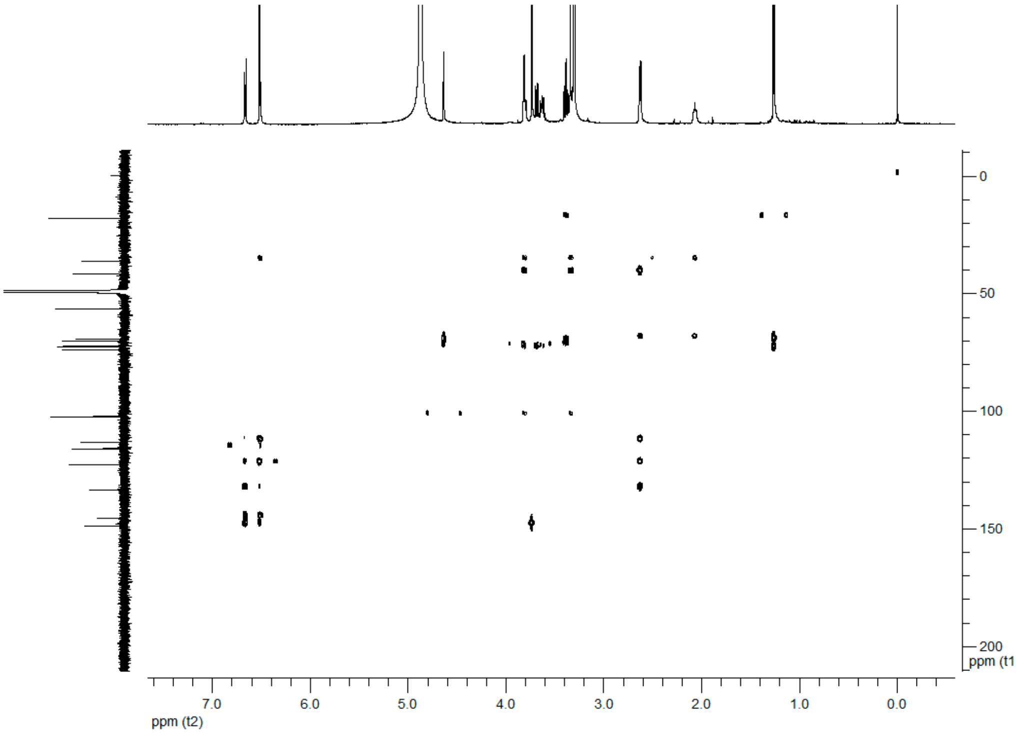

Figure S29. HMBC Spectrum of difengpioside D (**4**).

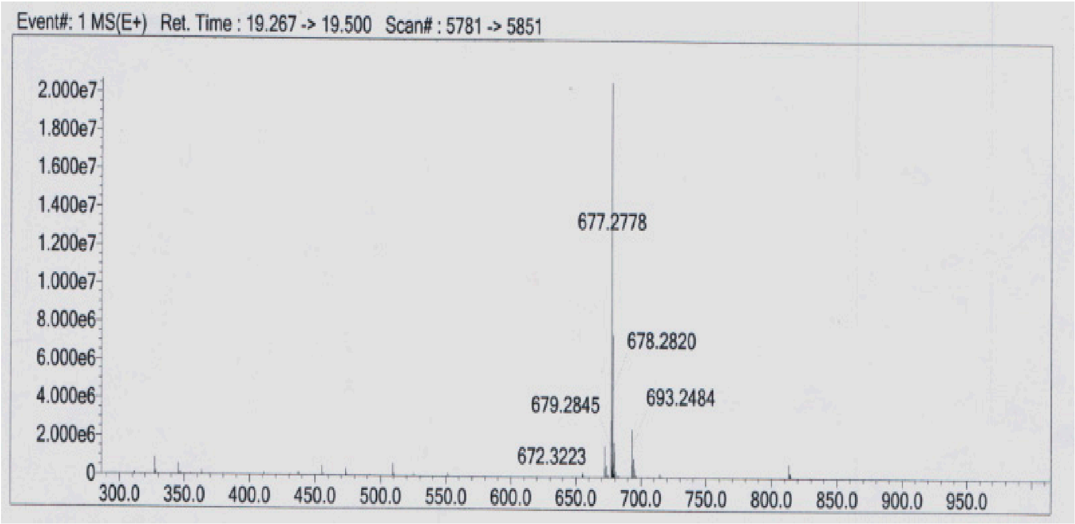

Figure S30. Positive HRESIMS spectrum of difengpioside D (**4**).

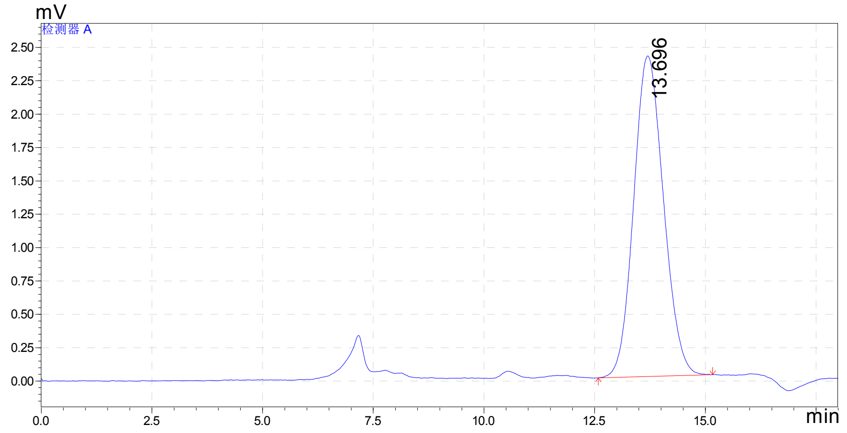

Figure S31. HPLC Analysis of the sugar of difengpioside D (**4**). Top: L-rhamnose; Below: sugar of compound **4**.

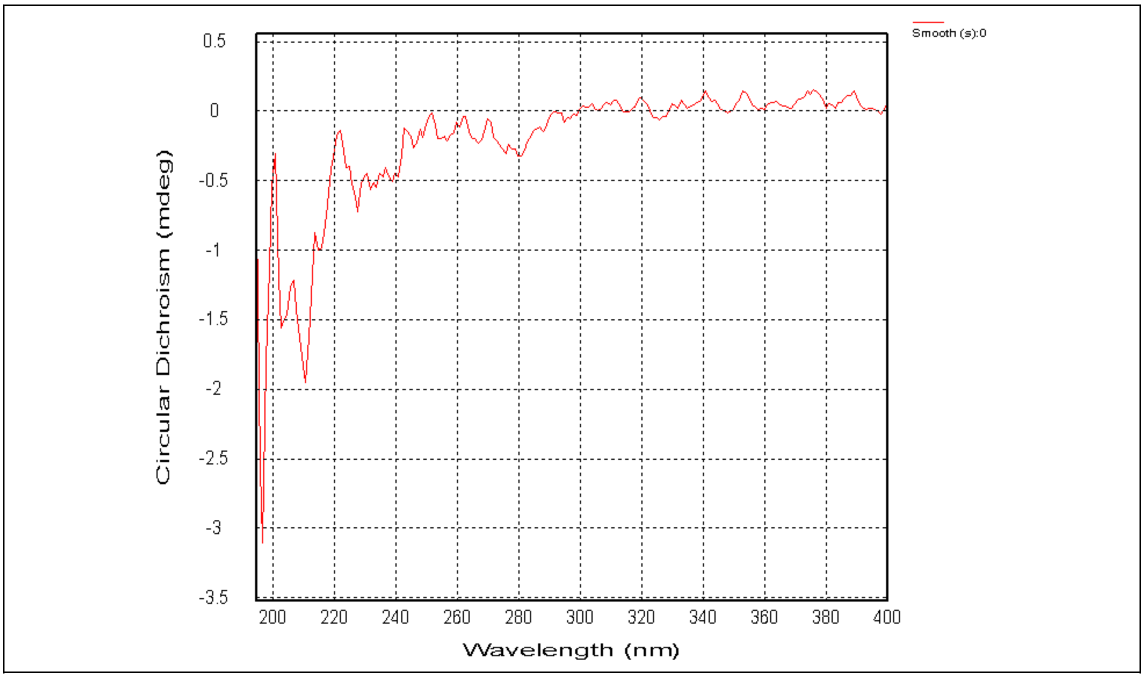

Figure S32. CD Spectrum of difengpioside D (**4**) in MeOH.
